# Deep learning for photoacoustic tomography from sparse data

**DOI:** 10.1080/17415977.2018.1518444

**Published:** 2018-09-11

**Authors:** Stephan Antholzer, Markus Haltmeier, Johannes Schwab

**Affiliations:** Department of Mathematics, University of Innsbruck, Innsbruck, Austria

**Keywords:** Photoacoustic tomography, sparse data, image reconstruction, deep learning, convolutional neural networks, inverse problems, 92C55, 45Q05, 65R32

## Abstract

The development of fast and accurate image reconstruction algorithms is a central aspect of computed tomography. In this paper, we investigate this issue for the sparse data problem in photoacoustic tomography (PAT). We develop a direct and highly efficient reconstruction algorithm based on deep learning. In our approach, image reconstruction is performed with a deep convolutional neural network (CNN), whose weights are adjusted prior to the actual image reconstruction based on a set of training data. The proposed reconstruction approach can be interpreted as a network that uses the PAT filtered backprojection algorithm for the first layer, followed by the U-net architecture for the remaining layers. Actual image reconstruction with deep learning consists in one evaluation of the trained CNN, which does not require time-consuming solution of the forward and adjoint problems. At the same time, our numerical results demonstrate that the proposed deep learning approach reconstructs images with a quality comparable to state of the art iterative approaches for PAT from sparse data.

## 1. Introduction

Deep learning is a rapidly emerging research area that yields significantly improved performance of many pattern recognition and machine learning applications [1,2]. Deep learning algorithms make use of special artificial neural network designs for representing a nonlinear input to output map together with optimization procedures for adjusting the weights of the network during the training phase. Deep learning techniques are currently the state of the art for visual object recognition, natural language understanding or applications in other domains such as drug discovery or biomedical image analysis (see, for example, [3–10] and the references therein).

Despite its success in various scientific disciplines, in image reconstruction deep learning research appeared only very recently (see [11–17]). In this paper, we develop a deep learning framework for image reconstruction in photoacoustic tomography (PAT). To concentrate on the main ideas we restrict ourselves to the sparse data problem in PAT in a circular measurement geometry. Our approach can be extended to an arbitrary measurement geometry in arbitrary dimension. Clearly, the increased dimensionality comes with an increased computational cost. This is especially the case for the training of the network which, however, is done prior to the actual image reconstruction.

### 1.1. PAT and the sparse sampling problem

**Figure 1.**
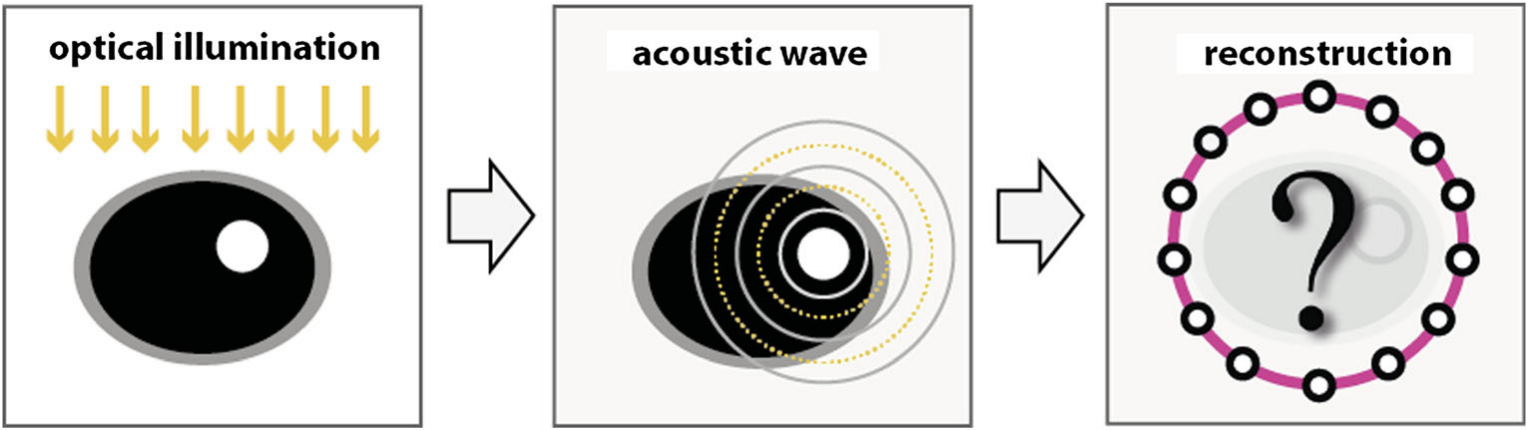
Basic principle of PAT. An object is illuminated with a short optical pulse (left) that induces an acoustic pressure wave (middle). The pressure signals are recorded outside of the object, and are used to recover an image of the interior (right).

PAT is a non-invasive coupled-physics biomedical imaging technology which beneficially combines the high contrast of pure optical imaging with the high spatial resolution of ultrasound imaging [18–21]. It is based on the generation of acoustic waves by illuminating a semi-transparent biological or medical object with short optical pulses. The induced time dependent acoustic waves are measured outside of the sample with acoustic detectors, and the measured data are used to recover an image of the interior (see Figure 1). High spatial resolution in PAT can be achieved by measuring the acoustic data with high spatial and temporal sampling rate [22,23]. While temporal samples can be easily collected at or above the Nyquist rate, the spatial sampling density is usually limited [24–29]. In fact, each spatial measurement requires a separate sensor and high quality detectors are often costly. Moving the detector elements can increase the spatial sampling rate, but is time consuming and also can introduce motion artefacts. Therefore, in actual applications, the number of sensor locations is usually small compared to the desired resolution which yields to a so-called sparse data problem.

Applying standard algorithms to sparse data yields low-quality images containing severe undersampling artefacts. To some extent, these artefacts can be reduced by using iterative image reconstruction algorithms [30–36] which allow to include prior knowledge such as smoothness, sparsity or total variation (TV) constraints [37–42]. These algorithms tend to be time consuming as the forward and adjoint problems have to be solved repeatedly. Further, iterative algorithms have their own limitations. For example, the reconstruction quality strongly depends on the used a-priori model about the objects to be recovered. For example, TV minimization assumes sparsity of the gradient of the image to be reconstructed. Such assumptions are often not strictly satisfied in real world scenarios which again limits the theoretically achievable reconstruction quality.

To overcome the above limitations, in this paper we develop a new reconstruction approach based on deep learning that comes with the following properties: (i) image reconstruction is efficient and non-iterative; (ii) no explicit a-priori model for the class of objects to be reconstructed is required; (iii) it yields a reconstruction quality comparable to (or even outperforming) existing methods for sparse data. Note that instead of providing an explicit a-priori model, the deep learning approach requires a set of training data and the CNN itself adjusts to the provided training data. By training the network on real word data, it thereby automatically creates a model of the considered PAT images in an implicit and data driven manner. While training of the CNN again requires time-consuming iterative minimization, we stress that training is performed independently of the particular investigated objects and prior to the actual image reconstruction. Additionally, if the time resources available for training a new network are limited, then one can use weights learned on one data set as good starting value for training the weights in the new network.

### 1.2. Proposed deep learning approach

**Figure 2.**
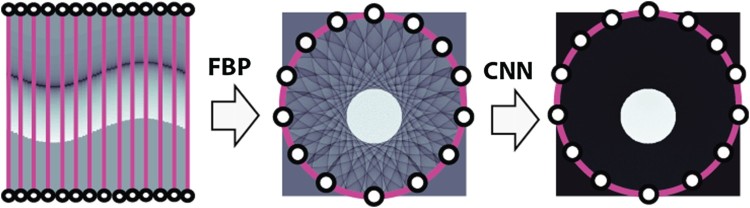
Illustration of the proposed network for PAT image reconstruction. In the first step, the FBP algorithm (or another standard linear reconstruction method) is applied to the sparse data. In a second step, a deep CNN is applied to the intermediate reconstruction which outputs an almost artefact-free image. This may be interpreted as a deep network with the FBP in the first layer and the CNN in the remaining layers.

Our reconstruction approach for the sparse data problem in PAT uses a deep convolutional neural network (CNN) in combination with any linear reconstruction method as preprocessing step. Essentially, it consists of the following two steps (see Figure 2):

1. In the first step, a linear PAT image reconstruction algorithm is applied, which yields an approximation of the original object including under-sampling artefacts.
2. In the second step, a deep CNN is applied to map the intermediate reconstruction from (D2) to an artefact-free final image.

Note that the above two-stage procedure can be viewed as a single deep neuronal network that uses the linear reconstruction algorithm in (D1) for the first layer, and the CNN in (D2) for the remaining layers.

Step (D1) can be implemented by any standard linear reconstruction algorithm including filtered backprojection (FBP) [43–49], Fourier methods [50–54], or time reversal [55–58]. In fact, all these methods can be implemented efficiently using at most $O(d^{3})$ floating point operations (FLOPS) for reconstructing a high-resolution image on an d×d grid. Here *d* is number spatial discretization points along one dimension of the reconstructed image. The CNN applied in step (D2) depends on weights that are adjusted using a set of training data to achieve artefact removal. The weights in the CNN are adjusted during the so-called training phase which is performed prior to the actual image reconstruction [1]. In our current implementation, we use the U-net architecture originally designed in [59] for image segmentation. Application of the trained network for image reconstruction is fast. One application of the U-net requires $O(F^{2}Ld^{2})$ FLOPS, where *F* is the number of channels in the first convolution and *L* describes the depth of the network. Typically, $F^{2}L$ will be in the order of *d* and the number of FLOPS for evaluating the CNN is comparable to the effort of performing an FBP reconstruction. Moreover, evaluation of the CNN can easily be parallelized, which further increases numerical performance. On the other hand, iterative reconstruction algorithms tend to be slower as they require repeated application of the PAT forward operator and its adjoint.

To the best of our knowledge, this is the first paper using deep learning or neural networks for image reconstruction in PAT. Related approaches applying CNNs for different medical imaging technologies including computed tomography (CT) and magnetic resonance imaging (MRI) appeared recently in [11–17]. The author of [14] shares his opinions on deep learning for image reconstruction. In [13], deep learning is applied to imaging problems where the normal operator is shift invariant; PAT does not belong to this class. A different learning approach for addressing the limited view problem in PAT is proposed in [60]. The above references show that a significant amount of research has been done on deep learning for CT and MRI image reconstruction (based on inverse Radon and inverse Fourier transforms). Opposed to that, PAT requires inverting the wave equation, and our work is the first paper that uses deep learning and CNNs for PAT image reconstruction and inversion of the wave equation.

### 1.3. Outline

The rest of this paper is organized as follows. In Section 2 we review PAT and discuss the sparse sampling problem. In Section 3 we describe the proposed deep learning approach. For that purpose, we discuss neural networks and present CNNs and the U-net actually implemented in our approach. Details on the numerical implementation and numerical results are presented in Section 4. The paper concludes with a short summary and outlook given in Section 5.

## 2. Photoacoustic tomography

As illustrated in Figure 1, PAT is based on generating an acoustic wave inside some investigated object using short optical pulses. Let us denote by $h:R^{d}\to R$ the initial pressure distribution which provides diagnostic information about the patient and which is the quantity of interest in PAT. Of practical relevance are the cases *d*=2,3 (see [20,61,62]). For keeping the presentation simple and focusing on the main ideas we only consider the case of *d*=2. Among others, the two-dimensional case arises in PAT with so called integrating line detectors [20,43]. Extensions to three spatial dimensions are possible by using the FBP algorithm for 3D PAT [45] in combination with the 3D U-net designed in [63]. Further, we restrict ourselves to the case of a circular measurement geometry, where the acoustic measurements are made on a circle surrounding the investigated object. In general geometry, one can use the so-called universal backprojection formula [47,49] that is exact for general geometry up to an additive smoothing term [47]. In this case, the CNN can be used to account for the under-sampling issue as well as to account for the additive smooth term. Such investigations, however, are beyond the scope of this paper.

### 2.1. PAT in circular measurement geometry

In two spatial dimensions, the induced pressure in PAT satisfies the following initial value problem for the 2D wave equation

(1) $$\begin{array}{ll} \partial_{t}^{2}p(x,t)-\Delta p(x,t)=0 & \mathrm{for}\;(x,t)\in R^{2}\times \left(0,\infty \right)\\ p(x,0)=h(x) & \mathrm{for}\;x\in R^{2}\\ \partial_{t}p(x,0)=0 & \mathrm{for}\;x\in R^{2}\;, \end{array}$$

where we assume a constant sound-speed that is rescaled to one. In the circular measurement geometry, the initial pressure *h* is assumed to vanish outside the disc $B_{R}:=\{x\in R^{2}|∥x∥<R\}$. Note that the solution of used forward wave equation (1) is, for positive times, equal to the causal solution of the wave equation with source term $\delta^{\prime }(t)h(x)$; see [64]. Both models (either with source term or with initial condition) are frequently used in PAT. The goal of PAT image reconstruction is to recover *h* from measurements of the acoustic pressure *p* made on the boundary $\partial B_{R}$.

In a complete data situation, PAT in a circular measurement geometry consist in recovering the function *h* from data

(2) $$(Ph)(z,t):=p(z,t)\;\mathrm{for}\;(z,t)\in \partial B_{R}\times [0,T]\;,$$

where *p* denotes the solution of (1) with initial data *h* and *T* is the final measurement time. Here complete data refers to data prior to sampling that are known on the full boundary $\partial B_{R}$ and up to times T≥2R. In such a case, exact and stable PAT image reconstruction is theoretically possible; see[32,65]. Several efficient methods for recovering *h* from complete data Ph are well investigated (see, for example, [43–58]). As an example, we mention the FBP formula derived in [44],

(3) $$h(r)=-\frac{1}{\pi R}\int_{\partial B_{R}}\int_{\left|r-z\right|}^{\infty }\frac{\left(\partial_{t}tPh)(z,t\right)}{\sqrt{t^{2}-|r-z{|}^{2}}}\;dt\;dS(z)\;.$$

Note that (3) requires data for all *t*>0; see [44, Theorem 1.4] for a related FBP formula that only uses data for *t*<2*R*. For the numerical results in this paper we truncate (3) at *t*=2*R*, in which situation all singularities of the initial pressure are contained in the reconstructed image and the truncation error is small.

### 2.2. Discretization and sparse sampling

In practical applications, the acoustic pressure Ph can only be measured with a finite number of acoustic detectors. The standard sampling scheme for PAT in circular geometry assumes uniformly sampled values

(4) $$\begin{array}{rl} p[m,\;\cdot \;] & :=Ph\left(z_{m},\;\cdot \;\right)\;,\;\mathrm{for}\;m=1,\ldots ,M\;, \end{array}$$

(5) $$\begin{array}{rl} \mathrm{with}\;z_{m} & :=\left[\begin{array}{c} R\cos\left(2\pi (m-1)/M\right)\\ R\sin\left(2\pi (m-1)/M\right) \end{array}\right]\;. \end{array}$$

Here p[m,⋅]:[0,T]→R is the signal corresponding to the *m*th detector, and *M* is the total number of detector locations. Of course, in practice also the signals p[m,⋅] have to be represented by discrete samples. However, temporal samples can easily be collected at a high sampling rate compared to the spatial sampling, where each sample requires a separate sensor.

**Figure 3.**
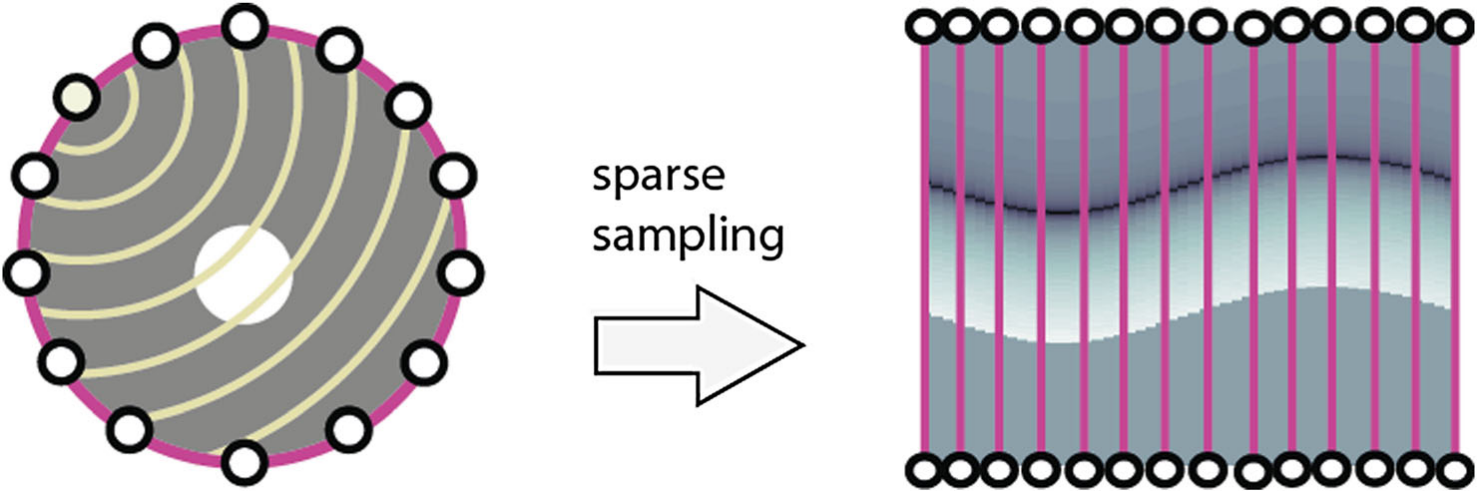
Sparse sampling problem in PAT in circular geometry. The induced acoustic pressure is measured at *M* detector locations on the boundary of the disc $B_{R}$ indicated by white dots in the left image. Every detector at location $z_{m}$ measures a time dependent pressure signal p[m,⋅], corresponding to a column in the right image.

In the case that a sufficiently large number of detectors is used, according to Shannon's sampling theory, implementations of full data methods yield almost artefact-free reconstructions (for a detailed analysis of sampling in PAT see [22]). As the fabrication of an array of detectors is demanding, experiments using integrating line detectors are often carried out using a single line detector, scanned on circular paths using scanning stages [66,67], which is very time consuming. Recently, systems using arrays of 64 parallel line detectors have been demonstrated [25,26]. To keep production costs low and to allow fast imaging the number *M* will typically be kept much smaller than advised by Shannon's sampling theory and one deals with highly under-sampled data. Therefore, one has to deal with a so-called sparse sampling problem (see Figure 3).

Due to the high frequency information contained in time, there is still hope to recover high-resolution images form spatially under-sampled data. For example, iterative algorithms, using TV minimization yield good reconstruction results from undersampled data (see [24,27,42,68]). However, such algorithms are quite time consuming as they require evaluating the forward and adjoint problem repeatedly (for TV typically at least several hundreds of times). Moreover, the reconstruction quality depends on certain a-priori assumptions on the class of objects to be reconstructed such as sparsity of the gradient. Image reconstruction with a trained CNN is direct and requires a smaller numerical effort compared to iterative methods. Further, it does not require an explicit model for the prior knowledge about the objects to be recovered. Instead, such a model is implicitly learned in a data-driven manner based on the training data by adjusting the weights of the CNN to the provided training data during the training phase.

## 3. Deep learning for PAT image reconstruction

Suppose that sparsely sampled data of the form (4), (5) are at our disposal. As illustrated in Figure 2, in our deep learning approach we first apply a linear reconstruction procedure to the sparsely sampled data $(p[m,\;\cdot \;]{)}_{m=1}^{M}$ which outputs a discrete image $X\in R^{d\times d}$. According to Shannon's sampling theory an aliasing free reconstruction requires M≥πd detector positions [22]. However, in practical applications we will have M≪d, in which case severe undersampling artefacts appear in the reconstructed image. To reduce these artefacts, we apply a CNN to the intermediate reconstruction which outputs an almost artefact-free reconstruction $Y\in R^{d\times d}$. How to implement such an approach is described in the following.

### 3.1. Image reconstruction by neural networks

The task of high-resolution image reconstruction can be formulated as supervised machine learning problem. In that context, the aim is finding a restoration function $\Phi :R^{d\times d}\to R^{d\times d}$ that maps the input image $X\in R^{d\times d}$ (containing undersampling artefacts) to the output image $Y\in R^{d\times d}$ which should be almost artefact-free. For constructing such a function Φ, one assumes that a family of training data $T:=(X_{n},Y_{n}{)}_{n=1}^{N}$ are given. Any training example $\left(X_{n},Y_{n}\right)$ consist of an input image $X_{n}$ and a corresponding artefact-free output image $Y_{n}$. The restoration function is constructed in such a way that the training error

(6) $$E(T;\Phi ):=\sum_{n=1}^{N}d(\Phi (X_{n}),Y_{n})$$

is minimized, where $d:R^{d\times d}\times R^{d\times d}\to R$ measures the error made by the function Φ on the training examples.

Particular powerful supervised machine learning methods are based on neural networks (NNs). In such a situation, the restoration function is taken in the form

(7) $$\Phi_{W}=(\sigma_{L}\circ W_{L})\circ \cdots \circ (\sigma_{1}\circ W_{1})\;,$$

where any factor $\sigma_{\ell }\circ W_{\ell }$ is the composition of a linear transformation (or matrix) $W_{\ell }\in R^{D_{\ell +1}\times D_{\ell }}$ and a nonlinearity $\sigma_{\ell }:R\to R$ that is applied component-wise. Here *L* denotes the number of processing layers, $\sigma_{\ell }$ are so called activation functions and $W:=(W_{1},\ldots ,W_{L})$ is the weight vector. Neural networks can be interpreted to consist of several layers, where the factor $\sigma_{\ell }\circ W_{\ell }$ maps the variables in layer ℓ to the variables in layer ℓ+1. The variables in the first layer are the entries of the input vector X and the variables in the last layer are the entries of the output vector Y. Note that in our situation we have an equal number of variables $D_{1}=D_{L+1}=d^{2}$ in the input and the output layer. Approximation properties of NNs have been analyzed, for example, in [69,70].

The entries of the weight vector W are called weights and are the variable parameters in the NN. They are adjusted during the training phase prior to the actual image reconstruction process. This is commonly implemented using gradient descent methods to minimize the training set error [1,71]

(8) $$E(T,W):=E(T,\Phi_{W})=\sum_{n=1}^{N}d\left(\Phi_{W}(X_{n}),Y_{n}\right).$$

The standard gradient method uses the update rule $W^{\left(k+1\right)}=W^{\left(k\right)}-\eta \;\nabla E(T,W^{\left(k\right)})$, where ∇E denotes the gradient of the error function in the second component and $W^{\left(k\right)}$ the weight vector in the *k*th iteration. In the context of neural networks, the update term is also known as error backpropagation. If the number of training examples is large, then the gradient method becomes slow. In such a situation, a popular acceleration is the stochastic gradient descent algorithm [1,71]. Here for each iteration a small subset $T^{\left(k\right)}$ of the whole training set is chosen randomly at any iteration and the weights are adjusted using the modified update formula $W^{\left(k+1\right)}=W^{\left(k\right)}-\eta \;\nabla E(T^{\left(k\right)},W^{\left(k\right)})$ for the *k*th iteration. In the context of image reconstruction similar acceleration strategies are known as ART or Kaczmarz type reconstruction methods [23,72,73]. The number of elements in $T^{\left(k\right)}$ is called batch size and *η* is referred to as the learning rate. To stabilize the iterative process, it is common to add a so-called momentum term $\beta \;(W^{\left(k\right)}-W^{\left(k-1\right)})$ with some nonnegative parameter *β* in the update of the *k*th iteration.

### 3.2. CNNs and the U-net

In our application, the inputs and outputs are high dimensional vectors. Such large-scale problems require special network designs, where the weight matrices are not taken as arbitrary matrices but take a special form reducing its effective dimensionality. When the input is an image, CNNs use such special network designs that are widely and successfully used in various applications [71,74]. A main property of CNNs is the invariance with respect to certain transformations of the input. In CNNs, the weight matrices are block diagonal, where each block corresponds to a convolution with a filter of small support and the number of blocks corresponds to the number of different filters (or channels) used in each layer. Each block is therefore a sparse band matrix, where the non-zero entries of the band matrices determine the filters of the convolution. CNNs are currently extensively used in image processing and image classification, since they outperform most comparable algorithms [1]. They are also the method of choice for the present paper.

**Figure 4.**
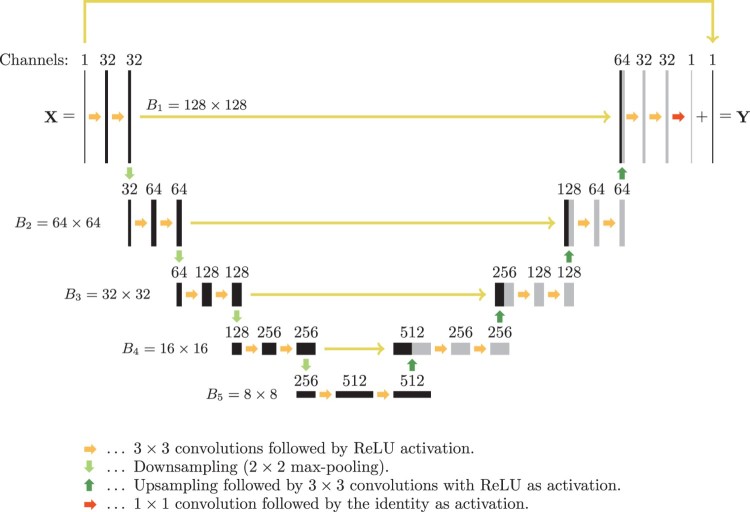
Architecture of the used CNN (U-net with residual connection). The number written above each layer denotes the number of convolution kernels (channels), which is equal to number of images in each layer. The numbers $B_{1},\ldots ,B_{5}$ denote the dimension of the images (the block sizes in the weight matrices), which stays constant in every row. The long horizontal arrows indicate direct connections with subsequent concatenation or summation for the upmost arrow.

There are various CNN designs that can differ in the number of layers, the form of the activation functions and the particular form of the weight matrices $W_{\ell }$. In this paper, we use a particular CNN based on the so-called U-net introduced in [59]. It has been originally designed for biomedical image segmentation and recently been used for low dose CT in [12,13]. The U-net is based on the so-called fully convolutional network used in reference [75]. Such network architectures employ multichannel filtering which means that the weight matrix in every layer consists of a family of multiple convolution filters followed by the rectified linear unit (ReLU) as activation function. The rectified linear unit is defined by ReLU⁡(x):=max{x,0}. As shown in [12], the residual images X−Y often have a simpler structure and are more accessible to the U-net than the original outputs. Therefore, learning the residuals and subtracting them from the inputs after the last layer is more effective than directly training for Y. Such an approach is followed in our implementation. The resulting deep neural network architecture is shown in Figure 4.

### 3.3. PAT using FBP combined with the U-net

We are now ready to present the proposed deep learning approach for PAT image reconstruction from sparse data, that uses the FBP algorithm as linear preprocessing step followed by the U-net for removing undersampling artefacts. Recall that we have given sparsely sampled data $(p[m,\;\cdot \;]{)}_{m=1}^{M}$ of the form (4), (5). A discrete high-resolution approximation $Y\in R^{d\times d}$ with d≫M of the original object is then reconstructed as follows.

1. Apply the FBP algorithm to p which yields an reconstruction $X\in R^{d\times d}$ containing undersampling artefacts.
2. Apply the U-net shown with residual connection in Figure 4 to X which yields an image $Y\in R^{d\times d}$ with significantly reduced undersampling artefacts.

The above two steps can also be combined to a single network with the FBP in the first layer and the U-net for the remaining layers. Note that the first step could also be replaced by another linear reconstruction methods such as time reversal and the second step by a different CNN. Such alternative implementations will be investigated in future studies. In this work, we use the FBP algorithm described in [44] for solving step (S1). It is based on discretizing the inversion formula (3) by replacing the inner and the outer integration by numerical quadrature and uses an interpolation procedure to reduce the numerical complexity. For details on the implementation we refer to [44,76].

A crucial ingredient in the above deep learning method is the adjustment of the actual weights in the U-net, which have to be trained on an appropriate training data set. For that purpose we construct training data $T=(X_{n},Y_{n}{)}_{n=1}^{N}$ by first creating certain phantoms $Y_{n}$. We then simulate sparse data by numerically implementing the well-known solution formula for the wave equation and subsequently construct $X_{n}$ by applying the FBP algorithm of [44] to the sparse data. For training the network we apply the stochastic gradient algorithm for minimizing the training set error (6), where we take the error measure *d* corresponding to the $\ell^{1}$-norm $∥Y{∥}_{1}=\sum_{i_{1},i_{2}=1}^{d}|Y[i_{1},i_{2}]|$.

## 4. Numerical realization

In this section, we give details on the numerical implementation of the deep learning approach and present reconstruction results under various scenarios.

### 4.1. Data generation and network training

For all numerical results presented below we use *d*=128 for the image size and take *R*=1 for the radius of the measurement curve. For the sparse data in (4) we use *M*=30 detector locations and discretize the pressure signals p[m,⋅] with 300 uniform samples in the time interval [0,2]. In our initial studies, we generate simple phantoms consisting of indicator functions of ellipses with support in the unit cube $[-1,1{]}^{2}\subseteq R^{2}$. For that purpose, we randomly generate solid ellipses *E* by sampling independent uniformly distributed random variables. The centres are selected uniformly in (−0.5,0.5) and the minor and major axes uniformly in (0.1,0.2).

For the training of the network on the ellipse phantoms we generate two different data sets, each consisting of *N*=1000 training pairs $(X_{n},Y_{n}{)}_{n=1}^{N}$. One set of training data corresponds to pressure data without noise and for the second data set we added random noise to the simulated pressure data. The outputs $Y_{n}$ consist of the sum of indicator functions of ellipses generated randomly as described above that are sampled on the 128×128 imaging grid. The number of ellipses in each training example is also taken randomly according to the uniform distribution on {1,…,5}. The input images are generated numerically by first computing the sparse pressure data using the solution formula for the wave equation and then applying the FBP algorithm to obtain $X_{n}$.

For actual training, we use the stochastic gradient descent algorithm with a batch size of one for minimizing (8). We train for 60 epochs which means we make 60 sweeps through the whole training dataset. We take $\eta =10^{-3}$ for the learning rate, include a momentum parameter β=0.99, and use the mean absolute error for the distance measure in (8). The weights in the *j*th layer are initialized by sampling the uniform distribution on $\left[-H_{\ell },H_{\ell }\right]$ where $H_{\ell }:=\sqrt{6}/\sqrt{D_{\ell }+D_{\ell +1}}$ and $D_{\ell }$ is the size of the input in layer ℓ. This initializer is due to Glorot and Bengio [77]. We use *F*=32 channels for the first convolution and the total number of layers is *L*=19.

### 4.2. Numerical results

**Figure 5.**
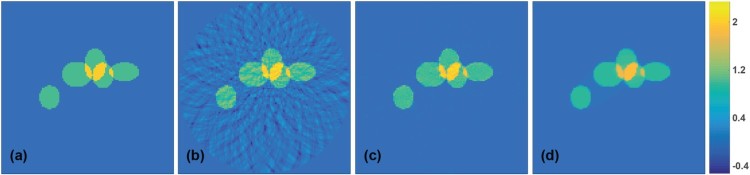
Results for simulated data (all images are displayed using the same colourmap). (a) Superposition of five ellipses as test phantom; (b) FBP reconstruction; (c) reconstruction using the proposed CNN; (d) TV reconstruction.

We first test the network trained above on a test set of 50 pairs (X,Y) that are generated according to the random model for the training data described above. For such random ellipse phantoms, the trained network is in all tested case able to almost completely eliminate the sparse data artefacts in the test images. Figure 5 illustrates such results for one of the test phantoms. Figure 5(a) shows the phantom, Figure 5(b) the result of the FPB algorithm which contains severe undersampling artefacts and Figure 5(c) the result of applying the CNN (right) which is almost artefact-free. The actual relative $\ell^{2}$-reconstruction error $∥Y_{\mathrm{CNN}}-Y{∥}_{2}/∥Y{∥}_{2}$ of the CNN reconstruction is 0.0087 which is much smaller than the relative error of FBP reconstruction which is 0.1811.

We also compared our trained network to penalized TV minimization [37,42]

(9) $$\frac{1}{2}∥p-P(Y){∥}_{2}^{2}+\lambda ∥Y{∥}_{\mathrm{TV}}\to \min_{Y}\;.$$

Here P is a discretization of the PAT forward operator using *M* detector locations and *d* spatial discretization points and $∥\;\cdot \;{∥}_{\mathrm{TV}}$ is the discrete TV. For the presented results, we take λ=0.002 and used the lagged diffusivity algorithm [78] with 20 outer and 20 inner iterations for numerically minimizing (9). TV minimization exploits the sparsity of the gradient as prior information and therefore is especially well suited for reconstructing sums of indicator functions and can be seen as state of the art approach for reconstructing such type of objects. As can be seen from the results  in Figure 5(d), TV minimization in fact gives very accurate results. Nevertheless, the deep learning approach yields comparable results in both cases. In terms of the relative $\ell^{2}$-reconstruction error, the CNN reconstruction even outperforms the TV reconstruction (compare with Table 1).

**Table 1.** Relative $\ell^{2}$-reconstruction errors for the four different test cases.

Phantom	FBP	TV	ELL	ELLn	SL	SLn
5 ellipses (exact)	0.1811	0.0144	0.0087	–	–	–
3 ellipses (noisy)	0.1952	0.0110	0.0051	0.0038	–	–
Shepp–Logan (exact)	0.3986	0.0139	0.1017	0.1013	0.0168	0.0186
Shepp–Logan (noisy)	0.3889	0.0154	0.1054	0.1027	0.0198	0.0206

Notes: Compared are FBP, TV, and the proposed CNN reconstruction trained on the class of ellipse phantoms without noise (ELL) and with noise (ELLn), as well as trained on a class containing Shepp–Logan type phantom without noise (SL) and with noise (SLn).

**Figure 6.**
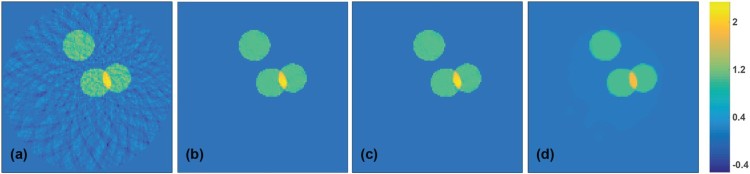
Results for noisy test data with 2% Gaussian noise added (all images are displayed using the same colourmap). (a) Reconstruction using the FBP algorithm; (b) reconstruction using the CNN trained without noise; (c) reconstruction using the CNN trained on noisy images; (d) TV reconstruction.

In order to test the stability with respect to noise we also test the network on reconstructions coming from noisy data. For that purpose, we added Gaussian noise with a standard deviation equal to 2% of the maximal value to simulated pressure data. Reconstruction results are shown in Figure 6. There we show reconstruction results with two differently trained networks. For the results shown in Figure 6(b) the CNN has been trained on the exact data, and for the results shown in Figure 6(c) it has been trained on noisy data. The reconstructions using each of the networks are again almost artefact-free. The reconstruction from the same data with TV minimization is shown in Figure 6(d). The relative $\ell^{2}$-reconstruction errors for all reconstructions are given in Table 1.

### 4.3. Results for Shepp–Logan type phantom

**Figure 7.**
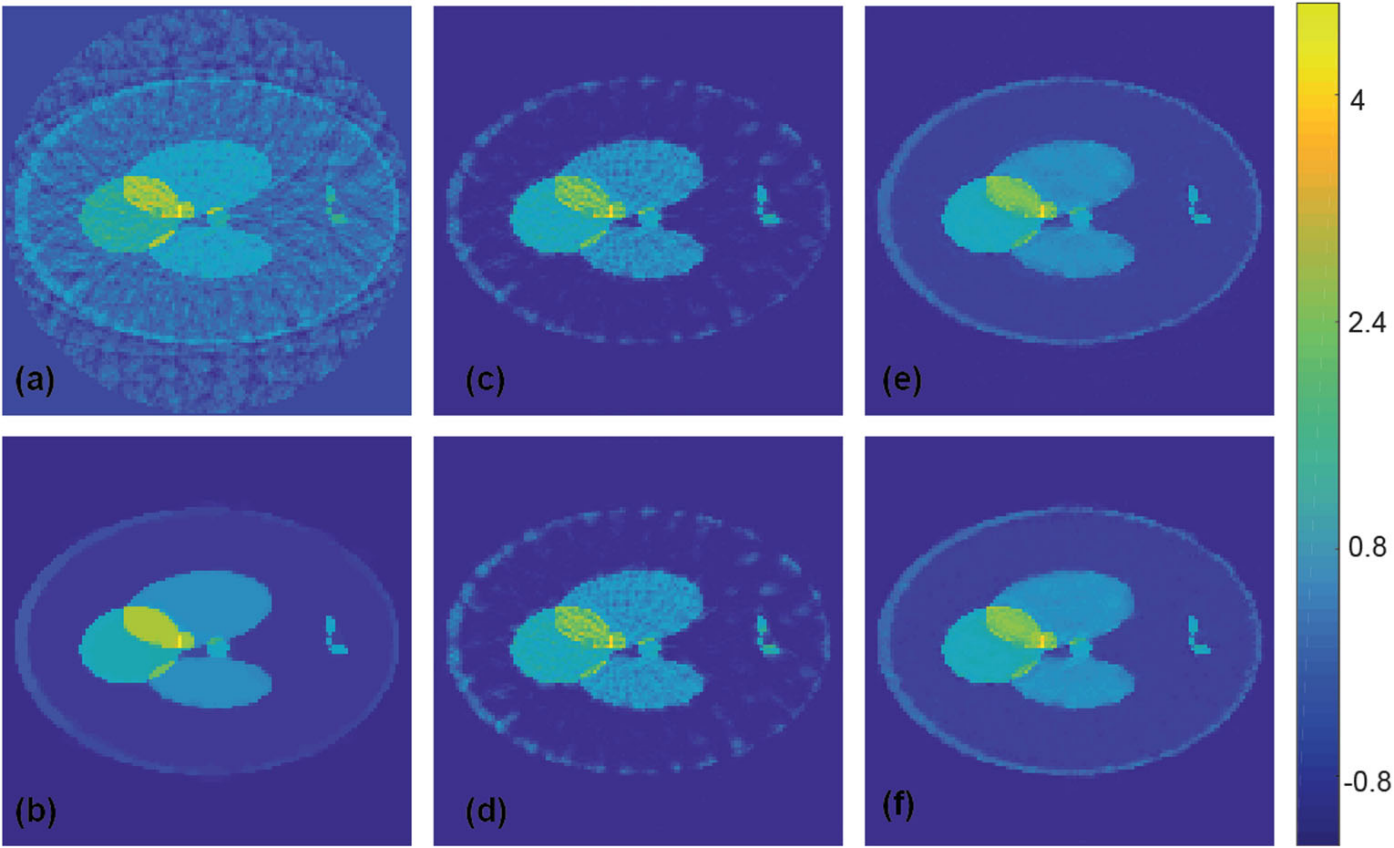
Reconstruction results for a Shepp–Logan type phantom using simultated data (all images are displayed using the same colourmap). (a) FBP reconstruction; (b) reconstruction using TV minimization. (c) Proposed CNN using wrong training data without noise added; (d) proposed CNN using wrong training data with noise added; (e) proposed CNN using appropriate training data without noise added; (f) proposed CNN using appropriate training data with noise added.

**Figure 8.**
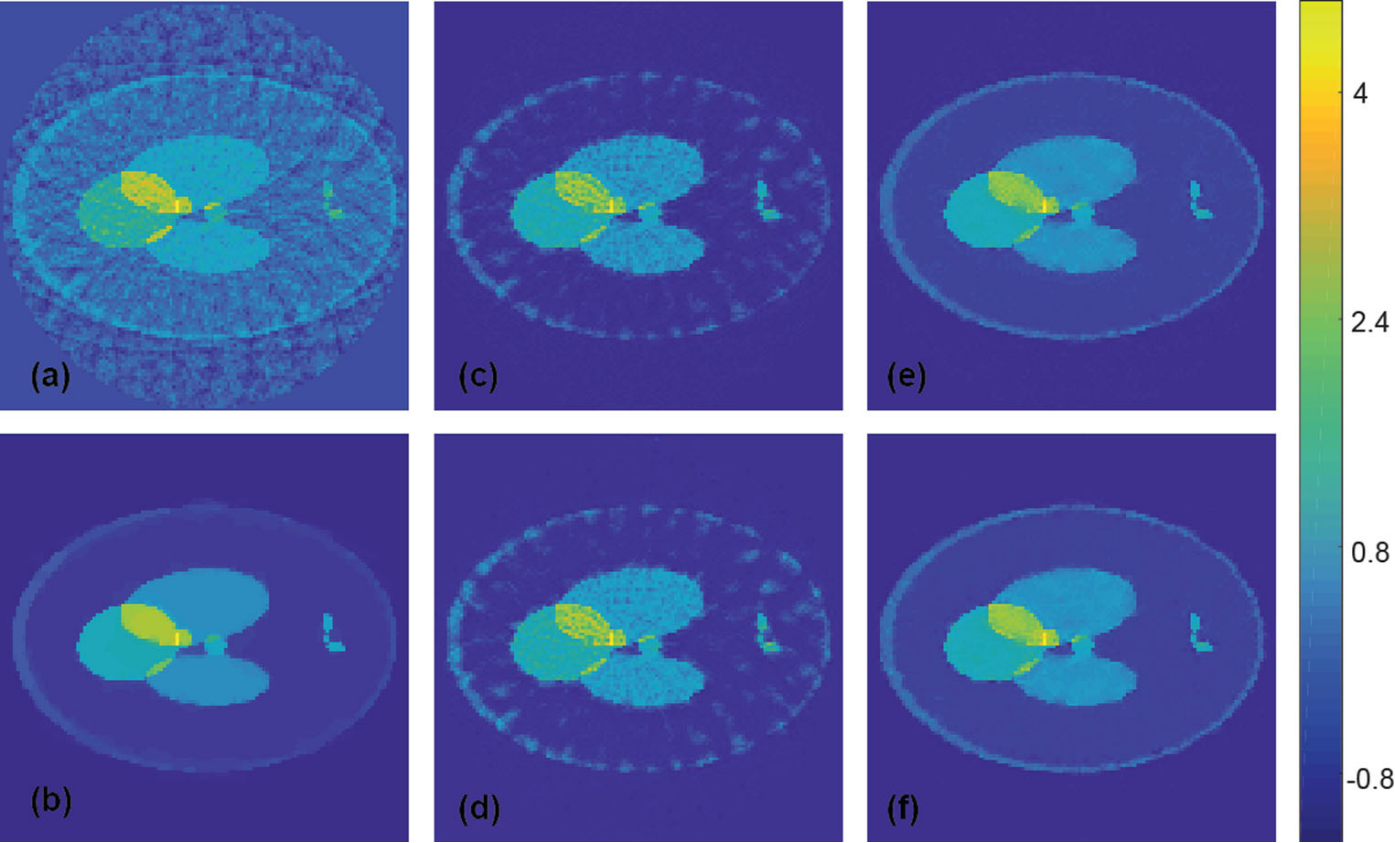
Reconstruction results for a Shepp–Logan type phantom from data with 2% Gaussian noise added (all images are displayed using the same colourmap). (a) FBP reconstruction; (b) reconstruction using TV minimization. (c) Proposed CNN using wrong training data without noise added; (d) proposed CNN using wrong training data with noise added; (e) proposed CNN using appropriate training data without noise added; (f) proposed CNN using appropriate training data with noise added.

In order to investigate the limitations of the proposed deep learning approach, we additionally applied the CNN (trained on the ellipse phantom class) to test phantoms where the training data are not appropriate (Shepp–Logan type phantoms). Reconstruction results for such a Shepp–Logan type phantom from exact data are shown in Figure 7, which compares results using FBP (Figure 7(a)), TV minimization (Figure 7(b)) and CNN improved versions using the ellipse phantom class without noise (Figure 7(c)) and with noise (Figure 7(d)) as training data. Figure 8 shows similar results for noisy measurement data with added Gaussian noise with a standard deviation equal to 2% of the maximal pressure value. As expected, this time the network does not completely remove all artefacts. However, despite the Shepp–Logan type test object has features not appearing in the training data, still many artefacts are removed by the network trained on the ellipse phantom class.

We point out, that the less good performance of CNN in Figures 7(a–d) and 8(a–d) is due to the non-appropriate training data and not due to the type of phantoms or the CNN approach itself. To support this claim, we trained additional CNNs on the union of 1000 randomly generated ellipse phantoms and 1000 randomly generated Shepp–Logan type phantoms. The Shepp–Logan type phantoms have position, angle, shape and intensity of every ellipse chosen uniformly at random under the side constraints that the support of every ellipse lies inside the unit disc. The results of the CNN trained on the new training data are shown in Figures 7(e,f) for exact measurement data and in Figure 8(e,f) for noisy measurement data. For both results we applied a CNN trained using training data without (e) and with noise (f). And indeed, when using appropriate training data including Shepp–Logan type phantoms, the CNN is again comparable to TV minimization. We see these results quite encouraging; future work will be done to extensively test the framework using a variety of training and test data sets, including real world data.

The relative $\ell^{2}$-reconstruction errors for all presented numerical results are summarized in Table 1.

### 4.4. Discussion

The above results demonstrate that deep learning-based methods are a promising tool to improve PAT image reconstruction. The presented results show that appropriately trained CNNs can significantly reduce under sampling artefacts and increase reconstruction quality. To further support this claim, in Table 2 we show the averaged relative $\ell^{2}$ reconstruction error for 100 Shepp–Logan type phantoms (similar to the ones in Figure 7). We see that even in the case where we train the network for the different class of ellipse shape phantoms, the error decreases significantly compared to FBP.

**Table 2.** Average error analysis.

Phantom	FBP	TV	ELL	ELLn	SL	SLn
Exact	0.2188	0.0140	0.1218	0.1078	0.0215	0.0213
Noisy	0.2374	0.0150	0.1290	0.1141	0.0256	0.0243

Notes: Relative $\ell^{2}$-reconstruction error averaged of over 100 Shepp–Logan type phantoms. Compared are FBP, TV, proposed CNN reconstruction trained on a class of ellipse phantoms without noise (ELL) and with noise (ELLn), as well as trained on a class of containing Shepp–Logan type phantom without SL and with noise (SLn).

Any reconstruction method for solving an ill-posed underdetermined problem, either implicitly or explicitly requires a-priori knowledge about the objects to be reconstructed. In classical variational regularization, a-priori knowledge is incorporated by selecting an element which has minimal (or at least small value) of a regularization functional among all objects consistent with the data. In the case of TV, this means that phantoms with small TV ($\ell^{1}$ norm of gradient) are reconstructed. On the other hand, in deep learning-based reconstruction methods a-priori knowledge is not explicitly prescribed in advance. Instead, the a-priori knowledge in encoded in the given training class and CNNs are trained to automatically learn the structure of desirable outputs. In the above results, the training class consists of piecewise constant phantoms which have small TV. Consequently, TV regularization is expected to perform well. It is therefore not surprising, that in this case TV minimization outperforms CNN-based methods. However, the CNN-based methods are more flexible in the sense that by changing the training set they can be adjusted to very different type of phantoms. For example, one can train the CNN for classes of experimental PAT data, where it may be difficult to find an appropriate convex regularizer.

### 4.5. Computational efforts

Application of the trained CNN for image reconstruction is non-iterative and efficient. In fact, one application of the used CNN requires $O(F^{2}Ld^{2})$ FLOPS, where *F* is the number of channels for the first convolution and *L* describes the depth of the network. Moreover, CNNs are easily accessible to parallelization. For high-resolution images, $F^{2}L$ will be in the order of *d* and therefore the effort for one evaluation of the CNN is comparable to effort of one evaluation of the PAT forward operator and its adjoint, which both require $O(d^{3})$ FLOPS. However, for computing the minimizer of (9) we have to repeatedly evaluate the PAT forward operator and its adjoint. In the examples presented above, for TV minimization we evaluated both operations 400 times, and therefore the deep learning image reconstruction approach is expected to be significantly faster than TV minimization or related iterative image reconstruction approaches.

For training and evaluation of the U-net we use the Keras software (see https://keras.io/), which is a high-level application programming interface written in Python. Keras runs on top of TensorFlow (see https://www.tensorflow.org/), the open-source software library for machine intelligence. These software packages allow an efficient and simple implementation of the modified U-net according to Figure 4. The filtered backprojection and the TV-minimization have been implemented in MATLAB. We perform our computations using an Intel Core i7-6850K CPU and a Nvidia Geforce 1080 Ti GPU. The training time for the CNN using the training set of 1000 ellipse phantoms has been 16 seconds per epoch, yields 16 minutes for the overall training time (using 60 epochs). For the larger mixed training data set (consisting of 1000 ellipse phantoms and 1000 Shepp–Logan type phantoms) one epoch requires 25 seconds. Recovering a single image requires 15 milliseconds for the FBP algorithm and 5 milliseconds for applying the CNN. The reconstruction time for the TV-minimization (with 20 outer and 20 inner iterations) algorithm has been 25 seconds. In summary, the total reconstruction time using the two-stage deep learning approach is 20 milliseconds, which is over 1000 times faster than the time required for the TV minimization algorithm. Of course, the reconstruction times strongly depend on the implementation of TV-minimization algorithm and the implementation of the CNN approach. However, any step in the iterative TV-minimization has to be evaluated in a sequential manner, which is a conceptual limitation of iterative methods. Evaluation of the CNN, on the other hand, is non-iterative and inherently parallel, which allows efficient parallel GPU computations.

## 5. Conclusion

In this paper, we developed a deep learning approach for PAT from sparse data. In our approach, we first apply a linear reconstruction algorithm to the sparsely sampled data and subsequently apply a CNN with weights adjusted to a set of training data. Evaluation of the CNN is non-iterative and has a similar numerical effort as the standard FBP algorithm for PAT. The proposed deep learning image reconstruction approach has been shown to offer a reconstruction quality similar to state of the art iterative algorithms and at the same time requires a computational effort similar to direct algorithms such as FBP. The presented numerical results can be seen as a proof of principle, that deep learning is feasible and highly promising for image reconstruction in PAT.

As demonstrated in Section 4 the proposed deep learning framework already offers a reconstruction quality comparable to state of the art iterative algorithms for the sparse data problem in PAT. However, as illustrated by Figures 7 and 8 this requires the PAT image to share similarities with the training data used to adjust the weights of the CNN. In future work, we will therefore investigate and test our approach under various real-world scenarios including realistic phantom classes for training and testing, different measurement geometries, and increased discretization sizes. In particular, we will also train and evaluate the CNNs on real world data.

Note that the results in the present paper assume an ideal impulse response of the acoustic measurement system. For example, this is appropriate for PAT using integrating optical line detectors, which have broad detection bandwidth; see [20]. In the case that piezoelectric sensors are used for acoustic detection, the limited bandwidth is an important issue that must be taken into account in the PAT forward model and the PAT inverse problem [79]. In particular, in this case, the CNN must also be trained to learn a deconvolution process addressing the impulse response function. Such investigations, as well as the application to real data, are beyond the scope of this paper and have been addressed in our recent work [80]. (Note that the presented paper has already been submitted much earlier, initially on 14 April 2017 and to IPSE on 1 July 2017.)

It is an interesting line of future research using other CNNs that may outperform the currently implemented U-net. We further work on the theoretical analysis of our proposal providing insight why it works that well, and how to steer the network design for further improving its performance and flexibility.

## References

[1] Goodfellow I, Bengio Y, Courville A. Deep learning. Cambridge (MA): MIT Press; 2016.

[2] LeCun Y, Bengio Y, Hinton G. Deep learning. Nature. 2015;521(7553):436–444. doi: 10.1038/nature14539 26017442

[3] Bahdanau D, Cho K, Bengio Y. Neural machine translation by jointly learning to align and translate. arXiv:1409.0473; 2014.

[4] Collobert R, Weston J, Bottou L, et al Natural language processing (almost) from scratch. J Mach Learn Res. 2011;12:2493–2537.

[5] Greenspan H, van Ginneken B, Summers RM. Guest editorial deep learning in medical imaging: overview and future promise of an exciting new technique. IEEE Trans Med Imaging. 2016;35(5):1153–1159. doi: 10.1109/TMI.2016.2553401

[6] Ioffe S, Szegedy C. Batch normalization: accelerating deep network training by reducing internal covariate shift. arXiv:1502.03167; 2015.

[7] Krizhevsky A, Sutskever I, Hinton GE. Imagenet classification with deep convolutional neural networks. Adv Neural Inf Process Syst. 2012;25:1097–1105.

[8] Litjens G, Kooi T, Ehteshami Bejnordi B, et al A survey on deep learning in medical image analysis. Med Image Anal 2017;41:60–88. 10.1016/j.media.2017.07.005 28778026

[9] Ma J, Sheridan RP, Liaw A, et al Deep neural nets as a method for quantitative structure–activity relationships. J Chem Inf Model. 2015;55(2):263–274. doi: 10.1021/ci500747n 25635324

[10] Wu R, Yan S, Shan Y, et al Deep image: scaling up image recognition. arXiv:1501.02876; 2015.

[11] Chen H, Zhang Y, Zhang W, et al Low-dose CT via convolutional neural network. Biomed Opt Express. 2017;8(2):679–694. doi: 10.1364/BOE.8.000679 28270976 PMC5330597

[12] Han Y, Yoo JJ, Ye JC. Deep residual learning for compressed sensing CT reconstruction via persistent homology analysis. Available from: arXiv:1611.06391; 2016.

[13] Jin KH, McCann MT, Froustey E, et al Deep convolutional neural network for inverse problems in imaging. arXiv:1611.03679; 2016. 10.1109/TIP.2017.2713099 28641250

[14] Wang G. A perspective on deep imaging. IEEE Access. 2016;4:8914–8924. doi: 10.1109/ACCESS.2016.2624938

[15] Wang S, Su Z, Ying L, et al Accelerating magnetic resonance imaging via deep learning. IEEE 13th international symposium on biomedical Imaging (ISBI); 2016. p. 514–517. 10.1109/ISBI.2016.7493320 PMC6839781 31709031

[16] Würfl T, Ghesu FC, Christlein V. Deep learning computed tomography. International conference on medical image computing and computer-assisted intervention; Springer: 2016. p. 432–440.

[17] Zhang H, Li L, Qiao K, et al Image prediction for limited-angle tomography via deep learning with convolutional neural network. arXiv:1607.08707; 2016.

[18] Beard P. Biomedical photoacoustic imaging. Interface Focus. 2011;1(4):602–631. doi: 10.1098/rsfs.2011.0028 22866233 PMC3262268

[19] Kruger RA, Lui P, Fang YR, et al Photoacoustic ultrasound (paus) – reconstruction tomography. Med Phys. 1995;22(10):1605–1609. doi: 10.1118/1.597429 8551984

[20] Paltauf G, Nuster R, Haltmeier M, et al Photoacoustic tomography using a Mach-Zehnder interferometer as an acoustic line detector. Appl Opt. 2007;46(16):3352–3358. doi: 10.1364/AO.46.003352 17514293

[21] Wang LV. Multiscale photoacoustic microscopy and computed tomography. Nat Photon. 2009;3(9):503–509. doi: 10.1038/nphoton.2009.157 PMC2802217 20161535

[22] Haltmeier M. Sampling conditions for the circular radon transform. IEEE Trans Image Process. 2016;25(6):2910–2919. doi: 10.1109/TIP.2016.2551364 27071168

[23] Natterer F. The mathematics of computerized tomographytte, volume 32 of Classics in applied mathematics. Philadelphia: SIAM; 2001.

[24] Arridge S, Beard P, Betcke M, et al Accelerated high-resolution photoacoustic tomography via compressed sensing. Phys Med Biol. 2016;61(24):8908. doi: 10.1088/1361-6560/61/24/8908 27910824

[25] Bauer-Marschallinger J, Felbermayer K, Bouchal K-D, et al Photoacoustic projection imaging using a 64-channel fiber optic detector array. Proc. San Francisco (CA): SPIE; 2015. p. 9323.

[26] Gratt S, Nuster R, Wurzinger G, et al 64-line-sensor array: fast imaging system for photoacoustic tomography. Proc SPIE. 2014;8943:894365.

[27] Guo Z, Li C, Song L, et al Compressed sensing in photoacoustic tomography in vivo. J Biomed Opt. 2010;15(2):021311–021311. doi: 10.1117/1.3381187 PMC2866258 20459233

[28] Rosenthal A, Ntziachristos V, Razansky D. Acoustic inversion in optoacoustic tomography: a review. Curr Med Imaging Rev. 2013;9(4):318–336. doi: 10.2174/15734056113096660006 24772060 PMC3996917

[29] Sandbichler M, Krahmer F, Berer T, et al A novel compressed sensing scheme for photoacoustic tomography. SIAM J Appl Math. 2015;75(6):2475–2494. doi: 10.1137/141001408

[30] Arridge SR, Betcke MM, Cox BT, et al On the adjoint operator in photoacoustic tomography. Inverse Problems. 2016;32(11):115012 (19pp). doi: 10.1088/0266-5611/32/11/115012

[31] Belhachmi Z, Glatz T, Scherzer O. A direct method for photoacoustic tomography with inhomogeneous sound speed. Inverse Problems. 2016;32(4):045005. doi: 10.1088/0266-5611/32/4/045005

[32] Haltmeier M, Nguyen LV. Analysis of iterative methods in photoacoustic tomography with variable sound speed. SIAM J Imaging Sci. 2017;10(2):751–781. doi: 10.1137/16M1104822 PMC10162777 37153495

[33] Huang C, Wang K, Nie L, et al Full-wave iterative image reconstruction in photoacoustic tomography with acoustically inhomogeneous media. IEEE Trans Med Imaging. 2013;32(6):1097–1110. doi: 10.1109/TMI.2013.2254496 23529196 PMC4114232

[34] Javaherian A, Holman S. A multi-grid iterative method for photoacoustic tomography. IEEE Trans Med Imaging. 2017;36(3):696–706. doi: 10.1109/TMI.2016.2625272 27834644

[35] Schwab J, Pereverzyev Jr S, Haltmeier M. A Galerkin least squares approach for photoacoustic tomography. arXiv:1612.08094; 2016.

[36] Wang K, Su R, Oraevsky AA, et al Investigation of iterative image reconstruction in three-dimensional optoacoustic tomography. Phys Med Biol. 2012;57(17):5399. doi: 10.1088/0031-9155/57/17/5399 22864062 PMC3532927

[37] Acar R, Vogel CR. Analysis of bounded variation penalty methods for ill-posed problems. Inverse Problems. 1994;10(6):1217–1229. doi: 10.1088/0266-5611/10/6/003

[38] Daubechies I, Defrise M, De Mol C. An iterative thresholding algorithm for linear inverse problems with a sparsity constraint. Comm Pure Appl Math. 2004;57(11):1413–1457. doi: 10.1002/cpa.20042

[39] Frikel J, Haltmeier M. Efficient regularization with wavelet sparsity constraints in photoacoustic tomography. Inverse Prob. 2018; 34:024006(28pp).

[40] Grasmair M, Haltmeier M, Scherzer O. Sparsity in inverse geophysical problems. In Freeden W, Nashed MZ, Sonar T, editors. Handbook of geomathematics. Berlin, Heidelberg: Springer; 2010. p. 763–784.

[41] Provost J, Lesage F. The application of compressed sensing for photo-acoustic tomography. IEEE Trans Med Imaging. 2009;28(4):585–594. doi: 10.1109/TMI.2008.2007825 19272991

[42] Scherzer O, Grasmair M, Grossauer H, et al Variational methods in imaging, volume 167 of Applied mathematical sciences. New York: Springer; 2009.

[43] Burgholzer P, Bauer-Marschallinger J, Grün H, et al Temporal back-projection algorithms for photoacoustic tomography with integrating line detectors. Inverse Problems. 2007;23(6):S65–S80. doi: 10.1088/0266-5611/23/6/S06

[44] Finch D, Haltmeier M, Rakesh Inversion of spherical means and the wave equation in even dimensions. SIAM J Appl Math. 2007;68(2):392–412. doi: 10.1137/070682137

[45] Finch D, Patch SK, Rakesh Determining a function from its mean values over a family of spheres. SIAM J Math Anal. 2004;35(5):1213–1240. doi: 10.1137/S0036141002417814

[46] Haltmeier M. Inversion of circular means and the wave equation on convex planar domains. Comput Math Appl. 2013;65(7):1025–1036. doi: 10.1016/j.camwa.2013.01.036

[47] Haltmeier M. Universal inversion formulas for recovering a function from spherical means. SIAM J Math Anal. 2014;46(1):214–232. doi: 10.1137/120881270

[48] Kunyansky LA. Explicit inversion formulae for the spherical mean Radon transform. Inverse Problems. 2007;23(1):373–383. doi: 10.1088/0266-5611/23/1/021

[49] Xu M, Wang LV. Universal back-projection algorithm for photoacoustic computed tomography. Phys Rev E. 2005;71(1):016706. 10.1103/PhysRevE.71.016706 15697763

[50] Agranovsky M, Kuchment P. Uniqueness of reconstruction and an inversion procedure for thermoacoustic and photoacoustic tomography with variable sound speed. Inverse Problems. 2007;23(5):2089–2102. doi: 10.1088/0266-5611/23/5/016

[51] Haltmeier M, Scherzer O, Zangerl G. A reconstruction algorithm for photoacoustic imaging based on the nonuniform FFT. IEEE Trans Med Imaging. 2009;28(11):1727–1735. doi: 10.1109/TMI.2009.2022623 19884063

[52] Jaeger M, Schüpbach S, Gertsch A, et al Fourier reconstruction in optoacoustic imaging using truncated regularized inverse k-space interpolation. Inverse Problems. 2007;23:S51–S63. doi: 10.1088/0266-5611/23/6/S05

[53] Kunyansky LA. A series solution and a fast algorithm for the inversion of the spherical mean Radon transform. Inverse Problems. 2007;23(6):S11–S20. doi: 10.1088/0266-5611/23/6/S02

[54] Xu Y, Xu M, Wang LV. Exact frequency-domain reconstruction for thermoacoustic tomography–II: cylindrical geometry. IEEE Trans Med Imaging. 2002;21:829–833. doi: 10.1109/TMI.2002.801171 12374320

[55] Burgholzer P, Matt GJ, Haltmeier M, et al Exact and approximate imaging methods for photoacoustic tomography using an arbitrary detection surface. Phys Rev E. 2007;75(4):046706. doi: 10.1103/PhysRevE.75.046706 17501015

[56] Hristova Y, Kuchment P, Nguyen L. Reconstruction and time reversal in thermoacoustic tomography in acoustically homogeneous and inhomogeneous media. Inverse Problems. 2008;24(5):055006(25pp). doi: 10.1088/0266-5611/24/5/055006

[57] Stefanov P, Uhlmann G. Thermoacoustic tomography with variable sound speed. Inverse Problems. 2009;25(7):075011 16. doi: 10.1088/0266-5611/25/7/075011

[58] Treeby BE, Cox BT. k-wave: MATLAB toolbox for the simulation and reconstruction of photoacoustic wave-fields. J Biomed Opt. 2010;15:021314. doi: 10.1117/1.3360308 20459236

[59] Ronneberge O, Fischer P, Brox T. U-net: convolutional networks for biomedical image segmentation. CoRR; 2015.

[60] Dreier F, Haltmeier M, Pereverzyev Jr S. Operator learning approach for the limited view problem in photoacoustic tomography. arXiv:1705.02698; 2017.

[61] Kuchment P, Kunyansky L. Mathematics of photoacoustic and thermoacoustic tomography. In: Otmar S, editor. Handbook math methods imaging. New York: Springer; 2011. p. 817–865.

[62] Xu M, Wang LV. Photoacoustic imaging in biomedicine. Rev Sci Instrum. 2006;77(4):041101(22pp). doi: 10.1063/1.2195024

[63] Çiçek O, Abdulkadir A, Lienkamp SS, et al 3d u-net: learning dense volumetric segmentation from sparse annotation. arXiv:1606.06650; 2016.

[64] Haltmeier M, Sandbichler M, Berer T, et al A sparsification and reconstruction strategy for compressed sensing photoacoustic tomography. J Acoust Soc Amer. 2018;141(6):3838(11pp). 10.1121/1.5042230 29960458

[65] Stefanov P, Uhlmann G. Thermoacoustic tomography with variable sound speed. Inverse Problems. 2009;25(7):075011 16. doi: 10.1088/0266-5611/25/7/075011

[66] Grün H, Berer T, Burgholzer P, et al Three-dimensional photoacoustic imaging using fiber-based line detectors. J Biomed Opt. 2010;15(2):021306–021308. doi: 10.1117/1.3381186 20459228

[67] Nuster R, Holotta M, Kremser C, et al Photoacoustic microtomography using optical interferometric detection. J Biomed Opt. 2010;15(2):021307–021307–6. doi: 10.1117/1.3333547 20459229

[68] Meng J, Wang LV, Liang D, et al In vivo optical-resolution photoacoustic computed tomography with compressed sensing. Opt Lett. 2012;37(22):4573–4575. doi: 10.1364/OL.37.004573 23164842

[69] Funahashi K. On the approximate realization of continuous mappings by neural networks. Neural Netw. 1989;2(3):183–192. doi: 10.1016/0893-6080(89)90003-8

[70] Hornik K. Approximation capabilities of multilayer feedforward networks. Neural Netw. 1991;4(2):251–257. doi: 10.1016/0893-6080(91)90009-T

[71] Bishop CM. Pattern recognition and machine learning. New York: Springer; 2006.

[72] De Cezaro A, Haltmeier M, Leitão A, et al On steepest-descent-Kaczmarz methods for regularizing systems of nonlinear ill-posed equations. Appl Math Comput. 2008;202(2):596–607.

[73] Gordon R, Bender R, Herman GT. Algebraic reconstruction techniques (ART) for three-dimensional electron microscopy and x-ray photography. J Theor Biol. 1970;29(3):471–481. doi: 10.1016/0022-5193(70)90109-8 5492997

[74] LeCun Y, Boser B, Denker JS, et al Backpropagation applied to handwritten zip code recognition. Neural Comput. 1989;1(4):541–551. doi: 10.1162/neco.1989.1.4.541

[75] Long J, Shelhamer E, Darrell T. Fully convolutional networks for semantic segmentation. Proceedings of the IEEE conference on computer vision and pattern recognition; Boston, USA; 2015. p. 3431–3440.

[76] Haltmeier M. A mollification approach for inverting the spherical mean Radon transform. SIAM J Appl Math. 2011;71(5):1637–1652. doi: 10.1137/110821561

[77] Glorot X, Bengio Y. Understanding the difficulty of training deep feedforward neural networks. Proceedings of the thirteenth international conference on artificial intelligence and statistics. Vol. 9. 2010; p. 249–256.

[78] Vogel CR, Oman ME. Iterative methods for total variation denoising. SIAM J Sci Comput. 1996;17:227–238. doi: 10.1137/0917016

[79] Paltauf G, Hartmair P, Kovachev G, et al Piezoelectric line detector array for photoacoustic tomography. Photoacoustics. 2017;8:28–36. doi: 10.1016/j.pacs.2017.09.002 28971019 PMC5619993

[80] Schwab J, Antholzer S, Nuster R, et al Real-time photoacoustic projection imaging using deep learning. arXiv:1801.06693; 2018.

